# From Narcissistic Personality Disorder to Frontotemporal Dementia: A Case Report

**DOI:** 10.3233/BEN-2011-0326

**Published:** 2011-05-23

**Authors:** Michele Poletti, Ubaldo Bonuccelli

**Affiliations:** ^1^Neurology UnitVersilia HospitalLido di Camaiore (LU)Italy; ^2^Neuroscience DepartmentUniversity of PisaPisaItaly

**Keywords:** Narcissistic personality disorder, frontotemporal dementia, risk factors

## Abstract

Premorbid personality characteristics could have a pathoplastic effect on behavioral symptoms and personality changes related to neurodegenerative diseases. Patients with personality disorders, in particular of the dramatic cluster, may present functional frontolimbic abnormalities. May these neurobiological vulnerabilities linked to a premorbid personality disorder predispose or represent a risk factor to subsequently develop a neurodegenerative disorder? Are subjects with personality disorders more at risk to develop a dementia than mentally healthy subjects? This topic is discussed presenting the clinical case of a patient who suffered of a probable Narcissistic Personality Disorder and subsequently developed a clinically diagnosed Frontotemporal Dementia.

## 1. Introduction

Neurodegenerative disorders give rise to behavioral and psychiatric symptoms as well as personality changes. The clinical experience suggests that, in mentally healthy subjects, the longstanding personality characteristics as a person's most distinctive features of all are likely to play a role in how someone with dementia cope with their increasing deficiencies: thus the premorbid personality characteristics could have a pathoplastic effect on the behavioral symptoms and the personality changes related to the neurodegenerative disease [1].

A slightly different question gives rise considering subjects with personality disorders, that usually present functional abnormalities and neurocognitive impairments. In this direction, the Borderline Personality Disorder (BPD) and the Antisocial Personality Disorder (APD) have been particularly studied [2]. Either adult subjects with diagnoses of BPD or APD show structural and functional frontolimbic anomalies [3–5]. May these neurobiological vulnerabilities linked to a personality disorder predispose or represent a risk factor to subsequently develop a neurodegenerative disorder during aging? Are subjects with personality disorders more at risk to develop a dementia than mentally healthy subjects? A brief literature review found only some case reports [6,7] and a lack of epidemiological data derived from samples of psychiatric patients with a personality disorder. A case report described a 46-year old woman with a diagnosis of BPD that developed clinical symptoms compatible with a co-existing diagnosis of Frontotemporal Dementia [6]; a case report described a 59-year old woman with a diagnosis of BPD that underwent clinical examination because of the fear of a dementia onset, but whose performances in neuropsychological tasks excluded the presence of a neurodegenerative disease [7].

In this paper we report the case of a patient who suffered of a probable Personality Disorder and subsequently developed a Frontotemporal Dementia.

ISSN 0953-4180/11/$27.50 © 2011 – IOS Press and the authors. All rights reserved

## 2. Case report

U.C., a 73 year-old retired man, who worked as chemical engineer in electronic industries, underwent our attention in 2008 for the appearance of behavioral disturbances, particularly disinhibition and aggressiveness. During a clinical interview with the patient and his wife, a pattern of abnormal behaviors and temperamental traits emerged; this pattern characterized the patient in his adulthood, since he began to work after his graduation. He reported that since he was a young adult, he began to study several law textbooks in order to be able to denounce all those individuals (politicians, officers, professors, freelancers) societies or institutions (private companies, public companies, local councils) that he deemed to have somehow broken the law or not protected the rights of himself or of someone else. As a matter of fact, as confirmed by his wife, he reported to have prepared, along many decades of his adulthood, some thousands of lawsuits; these lawsuits caused many problems to U.C., but he never thought to stop his activity. He reported to have written an erotic version of a famous bestseller fiction book that was published some years before, a version that would have had a great success if published. He also reported to have a superior intelligence, higher than those of almost all persons he knew.

This behavioral and temperamental pattern was compatible with a retrospective DSM-IV-TR based diagnosis of a Narcissistic Personality Disorder (NPD), a personality disorder defined as a pervasive pattern of grandiosity, need for admiration, and a lack of empathy, beginning by early adulthood and present in a variety of contexts. How U.C. described himself and its “mission of justice” were suggestive of several of the clinical features that characterizes the NPD (see diagnostic criteria of the DSM-IV-TR [8]): a grandiose sense of self-importance; preoccupation with fantasies of unlimited success, power or brilliance; beliefs to be special and to be understood by people (or institutions) who are also “special” or of high status; request of excessive admiration; a sense of entitlement; interpersonally exploitation; a lack of empathy; arrogant and haughty behaviors [9]. The behavioral and temperamental pattern of U.C. could also be compatible with a retrospective DSM-IV-TR based diagnosis of Paranoid Personality Disorder (PPD), a personality disorder characterized by a pervasive distrust and suspiciousness of others, such that their motives are interpreted as malevolent, beginning by early adulthood and present in a variety of contexts [8].

His wife reported that during last year U.C. began to present an altered behavior, compared to his adult personality, characterized by hypersexuality, verbal aggressivity and disinhibition (for example, after he accidentally hit a tree with his car, he reversed the car and began to voluntarily hit the three many times until the car was almost a wreck). During our neurological examination U.C. was logorheic and verbally disinhibited, with several sexual jokes. Scores in the Mini Mental State Examination [10] and in the Frontal Assessment Battery [11] were in the normal range. Because of his insufficient collaboration, it was not possible to administer a complete neuropsychological examination and a Structured Clinical Interview Axis II Disorders (SCID-II) [12]. Even if not formally correct, a retrospective interview with his wife, conducted following SCID-II, confirmed that the adult temperamental traits and the behaviors of U.C. were compatible with the presence of a NPD, and probably also of a PPD.

Present behavioral characteristics of U.C. were evaluated by the Frontal Behavioral Inventory [13], reporting a score of 28/72 (20 positive behaviors, 8 negative behaviors): a score of 17 or higher on the Frontal Behavioral Inventory (FBI) is 100-percent sensitive and 63-percent specific for Frontotemporal Dementia, while a score of 27 or higher is 90-percent sensitive and 100-percent specific [13]. The Magnetic Resonance Imaging revealed a clinical picture of leukoencefalopathy while the $^{18}$F-FDG PET revealed a bilateral prefrontal hypometabolism, more marked on the right hemisphere, and a less marked mesial temporal hypometabolism, more marked on the right hemisphere (See Fig. 1). Behavioral symptoms of UC were suggestive of a diagnosis of a possible behavioral variant Frontotemporal Dementia (bvFTD) whose clinical features, as proposed by Neary et al. [14], include an insidious onset and gradual progression, an early decline in social interpersonal conduct, an early impairment of regulation of personal conduct, an early emotional blunting and an early loss of insight. Supportive behavioural diagnostic features include a decline in personal hygiene and grooming, apathy and disinhibition, mental rigidity and inflexibility, distractibility and impersistence, hyperorality and dietary changes, perseverative and stereotyped behaviors, utilization behavior [15,16]. Also the PET findings were suggestive of a possible bvFTD, in line with recent $^{18}$F-FDG-PET studies on FTD patients, that reported a prominent prefrontal hypometabolism in this clinical population [17–19].

## 3. Discussion

We felt this case to be interesting because of patient's confounding psychiatric symptoms and a possible pre-existing psychiatric disorder. The patient U.C. had displayed a number of narcissistic and probably paranoid traits since young adulthood, maybe contributing to the delay in the clinical referral and diagnosis of bvFTD. However, several key features of U.C. illness support a diagnosis of bvFTD, including his progressive behavioral changes, specially the positive behaviors evidenced by FBI scores, and the decreased bilateral metabolic activity in the temporal and prefrontal cortices on the $^{18}$F-FDG-PET.

This case-report 1) illustrates the difficulties faced by clinicians in recognizing a relatively rare neurodegenerative condition, such as bvFTD, when the clinical picture is complicated by pre-existing psychiatric symptoms; 2) as previously explained, it raises the question about a possible increased risk of psychiatric patients to develop a dementia. Our patient U.C., with a probable pre-existing NPD, developed a bvFTD. Even if no structural and functional neuroradiologic findings are available on subjects with NPD, the presence of common neurocognitive impairments across different dramatic personality disorders of the Cluster B suggests that probably also subjects with a NPD may present frontolimbic dysfunctions [2,20]. Resuming, the patient U.C. probably presented a frontolimbic dysfunction already in his adulthood and, when aging, developed a neurodegenerative disorder, in turn characterized by a frontolimbic dysfunction: are these clinical phenomena related? May the premorbid frontolimbic dysfunction be considered a neurobiological vulnerability for the subsequent development of a dementia? Or the psychopathological features compatible with the diagnosis of NPD were instead the first clinical symptoms of a slow progressive bvFTD, with an insidious onset?

This case-report does not permit to directly relate the development of a bvFTD dementia to the possible pre-existing personality disorder and its neurobiological bases. The lack of epidemiological studies on the incidence of neurodegenerative diseases in psychiatric patients, in particular in patients with personality disorders, suggests to explore this possible relationship in future studies. The majority of studies assessing the relationship between adult personality and the development of dementia adopted personality inventories not developed within a clinical approach (see for example [21,22]) Only two studies investigated the relationship between personality disorders and the development of dementia with a focus on personality disorders: a study reported that the development of Alzheimer's disease was particularly associated with Cluster A personality disorder traits (paranoid, schizoid, and schizotypal symptoms) in adulthood [23], while another study reported that traits of passivity, avoidance, alexithymia and obsessivity were associated to the subsequent development of a dementia [24]. Paradoxically, in this study the prevalence of narcissistic features was higher in controls than in patients that developed a dementia. However all these studies adopted a retrospective methodology to assess adult personality of subjects with dementia, but personality inventories are not developed for a retrospective use and this constitutes a strong methodological bias; the recall of own premorbid personality (if inventories are administered to patients) or the premorbid personality of relatives (if inventories are administered to caregivers) is potentially flawed and the use of the term 'premorbid' is actually improper [25]. This suggests that future studies about this topic should be longitudinal, assessing if subjects with personality disorders or other psychiatric disorders are more vulnerable to subsequently develop a dementia.

Finally, the case of U.C. also underlines the usefulness of a longitudinal assessment of cognitive functions in patients with personality disorders in order to detect as early as possible cognitive changes suggestive of a possible neurodegenerative process.
